# Rotavirus in developing countries: molecular diversity, epidemiological insights, and strategies for effective vaccination

**DOI:** 10.3389/fmicb.2023.1297269

**Published:** 2024-01-05

**Authors:** Asma Sadiq, Jadoon Khan

**Affiliations:** ^1^Department of Microbiology, University of Jhang, Jhang, Pakistan; ^2^Department of Allied and Health Sciences, IQRA University, Chak Shahzad Campus, Islamabad, Pakistan

**Keywords:** rotavirus, worldwide, middle-income, live-attenuated, effectiveness

## Abstract

Rotavirus (RV) causes the loss of numerous children’s lives worldwide each year, and this burden is particularly heavy in low- and lower-middle-income countries where access to healthcare is limited. RV epidemiology exhibits a diverse range of genotypes, which can vary in prevalence and impact across different regions. The human genotypes that are most commonly recognized are G1P[8], G2P[4], G3P[8], G4P[8], G8P[8], G9P[8], and G12P[8]. The diversity of rotavirus genotypes presents a challenge in understanding its global distribution and developing effective vaccines. Oral, live-attenuated rotavirus vaccines have undergone evaluation in various contexts, encompassing both low-income and high-income populations, demonstrating their safety and effectiveness. Rotavirus vaccines have been introduced and implemented in over 120 countries, offering an opportunity to assess their effectiveness in diverse settings. However, these vaccines were less effective in areas with more rotavirus-related deaths and lower economic status compared to wealthier regions with fewer rotavirus-related deaths. Despite their lower efficacy, rotavirus vaccines significantly decrease the occurrence of diarrheal diseases and related mortality. They also prove to be cost-effective in regions with a high burden of such diseases. Regularly evaluating the impact, influence, and cost-effectiveness of rotavirus vaccines, especially the newly approved ones for worldwide use, is essential for deciding if these vaccines should be introduced in countries. This is especially important in places with limited resources to determine if a switch to a different vaccine is necessary. Future research in rotavirus epidemiology should focus on a comprehensive understanding of genotype diversity and its implications for vaccine effectiveness. It is crucial to monitor shifts in genotype prevalence and their association with disease severity, especially in high-risk populations. Policymakers should invest in robust surveillance systems to monitor rotavirus genotypes. This data can guide vaccine development and public health interventions. International collaboration and data sharing are vital to understand genotype diversity on a global scale and facilitate the development of more effective vaccines.

## Introduction

Rotavirus (RV) belongs to the Reoviridae family is one of the primary pathogen responsible for acute gastroenteritis in young children (Bibera et al., 2020). It is worth mentioning that by the time children reach the age of five, nearly every child across the world has encountered at least one instance of rotavirus-induced diarrhea (Shrestha et al., 2021). Rotavirus (RV) infection is responsible for about 258 million cases of infectious diarrhea in children under five worldwide (Troeger et al., 2018b). An estimated 122,000–215,000 diarrheal infant fatalities were attributed to RV between, 2013 and 2017 (Tate et al., 2016; Troeger et al., 2018b). According to a global health statistic from 2016, approximately 100 per 100,000 children die before reaching the age of five in the ten developing countries with the highest RV diarrheic burden (India, Pakistan, Kenya, Democratic Republic of the Congo, Niger, Angola, Ethiopia, Afghanistan, Nigeria, and Chad) (Tate et al., 2016).

Compared to high-income countries (HIC), children in low- and middle-income countries (LMIC) suffer significantly from diarrheal mortality (Troeger et al., 2018b; Omatola and Olaniran, 2022b). This is primarily due to factors such as heightened exposure to the pathogen, particularly in very young children, increased childhood comorbidities like HIV infection, bacterial gastroenteritis, or malnutrition, and insufficient access to preventive and treatment measures. These factors collectively account for the elevated mortality rates observed in these regions (Troeger et al., 2018a; Bibera et al., 2020).

In 2009, the World Health Organization (WHO) issued a recommendation that all children worldwide, particularly those in countries with high mortality rates linked to diarrhea, should be provided with vaccinations against Rotavirus (Tate et al., 2016). Currently, the WHO has granted prequalification status to four RV vaccines. Among them, Rotarix and RotaTeq have been used widely, While, Rotasiil, and Rotavac are available for use in India. Several countries that have introduced routine childhood vaccination against rotavirus have observed significant reductions in severe diarrhea and hospitalizations due to rotavirus disease (Patel et al., 2012). Moreover, some nations like Mexico, Brazil, and Panama have reported substantial declines in diarrhea-related mortality among children under the age of five, ranging from 22 to 50%, following the introduction of the vaccine (Richardson et al., 2010; do Carmo et al., 2011; Lanzieri et al., 2011; Bayard et al., 2012).

The main objective of this review article is to examine the complex scenario of rotavirus infections in developing countries. Our analysis encompasses an exploration of the distinct epidemiological trends associated with rotavirus infection in developing regions, the diversity of RVA genotypes, and an elucidation of the factors contributing to its prevalence. Additionally, we delve into the present status of rotavirus vaccine adoption and provide insights into potential future directions and recommendations. The information has the potential to steer the development of vaccines and public health interventions. Global comprehension of genotype diversity and the facilitation of more potent vaccine development hinge crucially on international collaboration and the sharing of data.

## RVA molecular biology

Rotavirus (RV) was initially identified in the 1950s when it was found in rectal swabs of monkeys. Subsequently, in the 1960s, the virus was observed in intestinal biopsies of mice using electron microscopy (Adams and Kraft, 1963). In 1973, Ruth Bishop and her team provided the first description of the virus in children who exhibited symptoms of gastroenteritis (Bishop et al., 1973). One year later, rotavirus was detected in significant amounts in fecal samples from hospitalized children suffering from acute nonbacterial gastroenteritis. This detection was achieved through direct thin-layer electron microscopy and immune electron microscopy (Bishop et al., 1974). When observed under the electron microscope, a viral particle displaying a wheel-like structure, measuring around 70 nanometers in diameter, was identified and subsequently named as “rotavirus.” The term “rotavirus” is derived from the Latin word “rota,” signifying a wheel (Flewett et al., 1974). The genus Rotavirus belongs to the family Sedoreoviridae within the Reovirales order (Matthijnssens et al., 2022). The rotavirus is a large, non-enveloped virus that has three concentric icosahedral capsid structures. Its diameter ranges from 65 to 75 nm. Two capsid proteins, VP4 and VP7, make up the outer layer. There is only a single kind of VP6 protein found in the intermediate layer. VP2 connected to VP1 (RNA-dependent RNA polymerase) and VP3 (viral capping enzyme) make up the internal core. The complete viral genome, which consists of 11 double-stranded RNA (dsRNA) segments, is enclosed within the internal core layer (Jampanil et al., 2023). The rotavirus genome codes for six nonstructural proteins (NSP1-NSP6) and six viral structural proteins (VP1-VP4, VP6, and VP7) (Desselberger, 2014). Based on antigenic and sequence variations of the two outer capsid proteins, VP7 and VP4 proteins, the rotavirus strains have been categorized into G (glycosylated) and P (protease-sensitive) genotypes, respectively, using a dual classification approach (Sadiq et al., 2018). Only a small number of G and P genotype combinations are primarily found in humans, despite the fact that 42 G genotypes and 58 P genotypes have been identified in both humans and animals globally (RCWG, 2023). RV has the capacity to generate novel antigen combinations of G-P genotypes through reassortment, with both these genotypes antigens being essential for the development of protective immunity, Consequently, it is vital to possess knowledge about the genetic variation of both G and P types (Gentsch et al., 2005).

The human genotypes that are most commonly recognized are G1P[8], G2P[4], G3P[8], G4P[8], G8P[8], G9P[8], and G12P[8] (Steger et al., 2019; Omatola and Olaniran, 2022b). G1P[8] predominates in North America, Europe, and Australia, constituting more than 70% of RV infections, in contrast to 30% in South America and Asia, and 23% in Africa. Other prevalent RV genotypes worldwide include G3P[8], G2P[4], and G4P[8], which, along with G1P[8], account for 50% of cases in Africa and a staggering 90% in Europe, North America, and Australia. Additionally, there have been identifications of G9 in combination with P [8], P [4], or P [6], along with an increased incidence of G12 strains (Bányai et al., 2012; Bibera et al., 2020).

The increasing availability of rotavirus sequence data has led to the establishment of a new rotavirus classification system by the Rotavirus Classification Working Group (RCWG). This system is based on the nucleotide sequence identities of each genome segment, organized in the order of Gx-P [x]-Ix-Rx-Cx-Mx-Ax-Nx-Tx-Ex-Hx, representing the genotypes of VP7-VP4-VP6-VP1-VP2-VP3-NSP1-NSP2-NSP3-NSP4-NSP5/6 (Matthijnssens et al., 2008b). Within rotavirus A, researchers have identified at least 42 G genotypes, 58 P genotypes, 32 I genotypes, 28 R genotypes, 24\u00B0C genotypes, 24 M genotypes, 39 A genotypes, 28 N genotypes, 28 T genotypes, 32 E genotypes, and 28 H genotypes from both humans and various animal species (RCWG, 2023). Rotavirus A has been found to circulate globally in humans across two genotype constellations: I1-R1-C1-M1-A1-N1-T1-E1-H1 (Wa-like) and I2-R2-C2-M2-A2-N2-T2-E2-H2 (DS-1-like) (Matthijnssens and Van Ranst, 2012). A common ancestor between the Wa-like strains and the porcine rotavirus has been proven, while some gene segments of the DS-1-like strains have been found to share an origin with the bovine rotavirus (Matthijnssens et al., 2008a). Another genotype (AU-1-like) that have been found in humans. G3-P [9]-I3-R3-C3-M3-A3-N3-T3-E3-H3 is also thought to have a common ancestor with rotavirus strains seen in cats and dogs (Nakagomi et al., 1990).

## RVA prevalence in developing countries

Rotavirus, the predominant cause of gastroenteritis, leads to infections and fatalities in all nations across the globe, with a more pronounced impact in developing countries (Du et al., 2022). Before the introduction of the rotavirus vaccine, rotavirus-induced diarrhea was the cause of death for approximately 527,000 children under the age of 5 worldwide on an annual basis. This accounted for roughly 40% of all deaths due to diarrhea and 5% of all deaths in the under-five age group (Parashar et al., 2009). It is worth noting that more than 90% of these deaths in 2013 occurred in 72 low and middle-income countries (Badur et al., 2019). The inclusion of the rotavirus vaccine in national immunization programs significantly reduced the burden of rotavirus-related disease. After vaccine implementation it is estimated that RVA is responsible for approximately 128,000 deaths in children under the age of five annually, with over 90% of these deaths occurring in resource-limited settings (Troeger et al., 2018b). In 2013, approximately 214,664 fatalities were linked to rotavirus infection within developing nations. These deaths were most concentrated in specific regions, namely Sub-Saharan Africa (with 121,000 deaths), Southern Asia (with 70,109 deaths), and Southeast Asia (with 10,765 deaths) (Tate et al., 2016), while other regions experienced a comparatively lower burden. Notably, nearly half of all global rotavirus-related deaths were concentrated in just four countries, namely India (with 47,100 deaths), Nigeria (with 30,800 deaths), Pakistan (with 14,700 deaths), and the Democratic Republic of Congo (with 13,526 deaths) (Hallowell et al., 2020).

The distribution of this disease burden varied significantly across different geographical regions. For example, in Europe, there were 75,000 to 150,000 cases of infant hospitalization due to acute gastroenteritis caused by rotavirus, while in Spain, the annual incidence of acute gastroenteritis associated with rotavirus ranged from 15.4 to 19.5 cases per 1,000 children up to 5 years and 20 cases per 1,000 children up to 3 years (Díez-Domingo et al., 2019). In the Eastern Mediterranean region, annual morbidity rates ranged from 0 to 112 per 100,000 with an average mortality rate of 39 per 10,000 per year (Omatola and Olaniran, 2022b). Generally, higher mortality rates from rotavirus gastroenteritis were observed in low-income countries (e.g., Afghanistan, Pakistan, Sudan, Yemen, and Somalia) compared to high-income countries (e.g., Saudi Arabia and Kuwait). However, both high- and low-income countries in the WHO-EMRO region had similar rates of hospital and health center visits for rotavirus gastroenteritis among children under five (Global Vaccine Action Plan, 2013; Badur et al., 2019).

The prevalence of RVA varies within and among developing countries. Some regions experience seasonal outbreaks, while others have a year-round occurrence. Factors such as climate, population density, and healthcare infrastructure influence these variations. Efforts to reduce RVA prevalence in developing countries include the introduction of RVA vaccines into national immunization programs. Vaccination has shown promising results in reducing the incidence and severity of RVA-associated diarrhea (Burnett et al., 2020). Over the past few decades, certain research findings have indicated a decrease in cases of rotavirus infections and associated fatalities. These reductions can be attributed to enhancements in safe water supplies, sanitation practices, and healthcare services. Additionally, advances in both prevention and treatment methods, notably the use of rotavirus vaccines, have contributed to this positive trend (Kawai et al., 2012; Lestari et al., 2020). Nevertheless, it is important to note that despite these improvements, the global burden of rotavirus infections remained substantial, with rotavirus remaining a prevalent cause of diarrhea worldwide (Troeger et al., 2018b).

## Epidemiology and diversity of RVA genotypes in developing countries

Rotavirus is the major cause of diarrheal morbidity and mortality in children under the age of five worldwide. The infection is usually acute and severe, with severe dehydration that necessitates hospitalization. Dehydration, if not treated promptly, can result in mortality, as is typical in underdeveloped nations (Omatola and Olaniran, 2022b). Risk factors for severe cases of rotavirus gastroenteritis include young age, preterm birth, low birthweight, malnutrition, socioeconomic disadvantage, impaired immunity, and co-infections with bacterial enteropathogens. However, it appears that transplacental maternal antibodies and breastfeeding may offer protection against rotavirus infections in children under 3 months of age (Schollin Ask, 2021). The primary transmission route is through the ingestion of contaminated fecal matter, typically resulting in an incubation period of 2–4 days.

The dominant genotype distribution of RV among the data collected from Southeast Asian countries has undergone changes, with the exception of Lao PDR and Malaysia. From 2009 to 2013, G1P[8] and G2P[4] were the prevailing genotypes. However, beginning in 2014, there was a shift toward less common and unusual genotypes, namely G3P[8], G8P[8], and G9P[8]. Additionally, the surveillance data revealed the presence of uncommon RV genotypes like G2P[8], G8P[6], G5P[19], G9P[4], G9P[6], and G1P7[5]. This diversity among RV isolates offers valuable insights into their evolution, which can result from factors such as point mutations, genetic rearrangements, reassortment events, and interspecies transmission (Gentsch et al., 2005; Kirkwood, 2010; Lestari et al., 2020). The diversity of circulating RV strains persists despite RV vaccination efforts, potentially leading to a rise in non-vaccine strains. Consequently, the continued circulation of strains with reduced vaccine effectiveness can eventually undermine the overall efficacy of the vaccine. In the Philippines, where the Rotarix® vaccine was introduced in July 2012, there has been a decrease in RVGE cases caused by the G1P[8] strain, while the prevalence of the G9P[8] strain has significantly increased (Leshem et al., 2014). The reason for high RVA genotype diversity in developing nations is due to conditions like overcrowding, shared water sources, and cohabitation of domestic animals and humans are prevalent, uncommon combinations of human G/P types are frequently reported. This is attributed to the increased likelihood of interspecies transmission of rotaviruses and reassortment event (Rojas et al., 2019). Consequently, unusual rotavirus genotypes like G1P[4], G2P[8], G9P[4], G12P[4], G8P[6], G8P[8], and G12P[6] have gained greater epidemiological significance in certain rural regions of Africa, Asia, and South America (Seheri et al., 2018; Rakau et al., 2021).

The epidemiological patterns of rotavirus disease vary between low- and lower-middle-income countries (LMICs) characterized by low rotavirus-related mortality and high-income countries (HICs) experiencing high rotavirus-related mortality. In HICs, rotavirus diarrhea tends to display a distinct seasonality during autumn and winter, whereas in LMICs, it is characterized by year-round transmission and a higher force of infection (Troeger et al., 2018b). Additionally, severe cases of rotavirus gastroenteritis predominantly affect younger infants and children in LMICs compared to HICs, rendering them more susceptible to rapid dehydration and fatality. A study comparing the incidence and severity of rotavirus diarrhea in sub-Saharan Africa and Europe revealed that children under 2 years old in Africa experience infections at an earlier age, with the peak incidence occurring around 5 months in regions with higher mortality rates, in contrast to approximately 20 months in areas with lower mortality rates (Steele et al., 2016). Furthermore, children in high-mortality countries often suffer multiple episodes of rotavirus diarrhea, and even after two to three infections, they do not develop complete immunity, as demonstrated by an Indian cohort study. This contrasts with observations in low-mortality countries, where two or three previous infections provided 57 and 79% protection against subsequent severe rotavirus gastroenteritis (Gladstone et al., 2011). Given these findings and the suboptimal performance of other oral vaccines like polio and cholera vaccines in resource-constrained settings, it was expected that rotavirus vaccines might have reduced efficacy in high-mortality settings. Consequently, it is crucial to assess both vaccine efficacy and effectiveness in low-income countries (LICs) and LMICs, as recommended by the World Health Organization (WHO) (Varghese et al., 2022).

It is crucial to take into account the variety of rotavirus strains, as this variability could potentially influence the performance and success of a vaccine in regions with a high disease burden (Hoshino and Kapikian, 2000). Rotaviruses are categorized based on the characterization of two viral proteins found on the outer capsid: VP7, which comprises the outer layer of the viral particle, and VP4, a spike protein responsible for facilitating the attachment and entry of the virus into host cells. Both of these viral proteins have been observed to stimulate the production of neutralizing antibodies in the host, and as a result, most vaccine development efforts have focused on them up to this point (Bishop et al., 1974; Clark et al., 2004).

The WHO’s worldwide rotavirus surveillance network and various regional networks have recorded a significant range of strains in Low-Income Countries (LICs) and Low- to Middle-Income Countries (LMICs) in contrast to High-Income Countries (HICs). Multiple global strain reviews have highlighted the presence of uncommon rotavirus strains, such as the VP4 P strains in Africa and South Asia. Furthermore, instances of rotavirus reassortant strains resulting from potential zoonotic infections and reassortment events have been frequently observed in Africa and Asia. These occurrences could pose challenges to the effectiveness of vaccines developed specifically for the more commonly found strains circulating among humans (Varghese et al., 2022).

Implementing a robust surveillance system enables the timely identification of emerging genotypes, allowing for the adaptation of vaccines to better match the circulating strains. This proactive approach not only helps maintain the effectiveness of immunization programs but also contributes to the global effort to reduce the burden of rotavirus-related diseases in vulnerable populations, especially in developing nations where healthcare resources may be limited.

## Exposure routes in emerging nations

**Figure 1:**
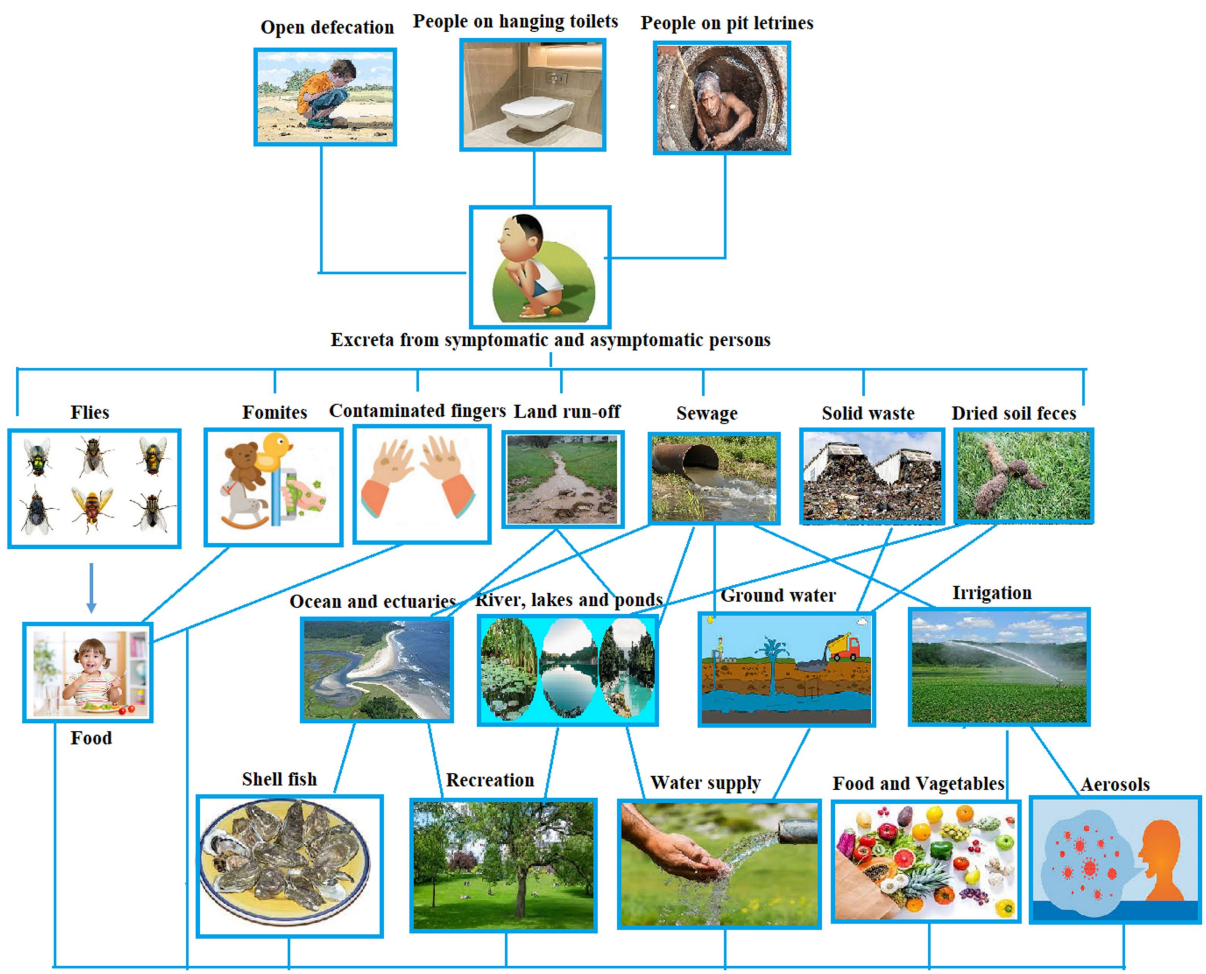
Pathways of rotavirus transmission (conceptualized based on Omatola and Olaniran, 2022b).

Rotavirus is transmitted primarily through the fecal-oral route (Figure 1). This viral infection, which mostly affects newborns and young children, is most commonly spread by direct person-to-person contact. The spread of the virus from feces to humans is primarily facilitated by environmental sources, including fluids, food, hands, and contaminated surfaces. This transmission can occur through interactions with the environment, either by humans or animals (Omatola and Olaniran, 2022b). Additionally, flies, as part of their natural behavior, can also contribute to the dissemination of rotavirus from feces. The virus is particularly prone to spreading among children and can be transmitted from infected children to close contacts. In individuals affected by the virus, the initial stage of the illness is usually characterized by more severe symptoms, which tend to become milder or even asymptomatic in some cases. Among adults, those with asymptomatic infections can still transmit the virus to close contacts (CDC, 2015).

The frequent exposure of susceptible children in daycare centers and family daycare homes makes these environments conducive to rotavirus transmission (CDC, 2015). Evidence of rotavirus has been found on various surfaces in homes and daycare centers, such as diaper disposal containers, toys, faucets, diaper changing areas, hand washing areas, and even in food preparation areas (Wilde et al., 1992). Children, in particular, have a tendency to put toys in their mouths or use them for teething, which can efficiently transmit rotavirus if the objects are contaminated. There have been reports of nosocomial outbreaks of rotavirus in healthcare settings, including a case in a pediatric oncology unit hospital linked to the sharing of toys among children (Rogers et al., 2000). Asymptomatic children shedding the virus tend to have lower viral levels, with intermittent shedding, compared to children with diarrhea. Both asymptomatic and symptomatic healthcare workers have been implicated in some outbreaks as sources of virus transmission (Omatola and Olaniran, 2022b).

Rotavirus is a highly resilient and widespread pathogen that can persist in the environment for weeks or even months without losing its infectivity if not properly disinfected. It can adhere to both porous (such as paper and cotton cloth) and nonporous (such as aluminum and latex) surfaces. The virus’s transmission and prevalence are facilitated by its low infectious dose (fewer than 100 viral particles), high concentration in feces (up to 10^12^ particles per gram), and extended period of viral shedding (Abad et al., 1994; CDC, 2023).

Fecal contamination of food, which is identified as an effective means of transmitting RV, typically occurs when contaminated water or inadequately treated sewage sludge and effluents are utilized for crop irrigation. It can also happen if food handlers neglect to maintain proper hand hygiene, resulting in foodborne illnesses (Division et al., 2005; Julian, 2016). Quiroz-Santiago et al. (2014) identified the presence of RVA in oysters and also documented its presence in 21.2% (7 out of 33) of vegetable samples such as celery, coriander, spinach, romaine lettuce, papaloquelite, and parsley that were brought to a Mexican market. Likewise, RVA was found in partially treated water (11.8%), irrigation water (14%), and the corresponding raw vegetable samples (1.7%) in Southern Africa (Van Zyl et al., 2006). Genotyping studies additionally revealed clinically significant strains of VP 7 (G) (G1, G2, G8, and G9) and VP4 (P) types (P [4], P [6], P [8], and P [9]). There have been multiple instances of foodborne rotavirus gastroenteritis outbreaks associated with contaminated food. Food items implicated in RV outbreaks have included crustaceans (Le Guyader et al., 2008), tuna and chicken sandwiches (CDC, 2000; Mizukoshi et al., 2014), salads (Fleet et al., 2020), and a potato stew (Mayr et al., 2009).

The literature has demonstrated the role of human fingers in the transmission of rotaviral infections through vehicles. Research has shown that infectious rotavirus (RV) particles, when placed on human fingers, can remain active for over 60 min without losing their infectivity, potentially leading to the contamination of surfaces when touched (Mayr et al., 2009).

Flies possess a natural inclination toward both fecal matter and food, which plays a crucial role as reservoirs for RV (Rotavirus) and other enteric pathogen transmissions (Julian, 2016). In the case of rotaviruses found in feces, they are typically acquired through direct contact with the fly’s outer body or by ingestion of the fecal matter. The contamination of various surfaces, including food items, objects (fomites), or the skin, can occur as a result of mechanical transmission. This transmission can happen through the transfer of RV from the fly’s outer body, regurgitation, or the deposition of fecal matter (Tan et al., 1997). The abundance of flies commonly observed in areas frequented by human activities, such as restaurants, food markets, fish markets, slaughterhouses, and hospitals, has been associated with a significant risk of viral transmission and subsequent infection when the pathogens are eventually ingested or come into contact with the mouth (Issa, 2019).

The rise in rotavirus transmission within developing nations is attributed to increased population growth and inadequate sanitation, particularly through the contamination of sewage or polluted river water (Omatola and Olaniran, 2022a). Additionally, the presence of rotavirus in drinking water that has undergone final treatment remains a persisting challenge (Van Zyl et al., 2006).

There has been a hypothesis regarding the possibility of rotavirus infection being transmitted through the air, which is based on factors such as the short incubation period (1–3 days), the swift seasonal transmission among populations, and the extensive scale of outbreaks. However, it should be noted that air borne transmission is not a confirmed or well-established mode of transmission for rotavirus. This mode of transmission however is not confirmed in humans (Omatola and Olaniran, 2022b).

## Rotavirus vaccines

Vaccination has been recognized as an effective approach for diminishing the likelihood of RV infections and significantly alleviating the impact of the disease. The management of diarrheal diseases caused by rotavirus primarily hinges on the utilization of live attenuated oral rotavirus vaccines, particularly in regions with elevated mortality rates (WHO, 2021) Several vaccines have been developed to prevent rotavirus gastroenteritis (RVGE), effectively safeguarding children against it. Within a decade of Ruth Bishop and her colleagues’ discovery of rotavirus in Melbourne, Australia in 1973, and the subsequent global recognition of this virus, the initial assessment of live-attenuated rotavirus vaccines in human subjects began (Bishop et al., 1974).

The first licensed rotavirus vaccine, known as RotaShield and developed by Wyeth Laboratories, Inc. in Marietta, PA, United States, received FDA approval in 1998. This approval followed confirmation of most of these findings through trials conducted in the USA, Finland, and Venezuela (Flores et al., 1987). The vaccine, created by the US National Institutes of Health (NIH), was a reassortant rhesus-rotavirus candidate expressing four of the most common outer capsid viral proteins to provide protection against various strains. However, approximately 9 months after its introduction in the USA, RotaShield was withdrawn from the market due to its association with intussusception, a rare side effect that is now recognized as being linked to multiple live-attenuated oral rotavirus vaccines, although not at the same level of risk as observed with RotaShield (Varghese et al., 2022). The World Health Organisation (WHO) has currently prequalified four rotavirus vaccines for global use, including Rotarix, RotaTeq Rotavac, and RotaSiil (Rota Council, 2019; Table 1).

**Table 1:** Properties of rotavirus vaccines that have been granted approval for use globally.

Vaccine name	Authorization for usage	WHO prequalification date	Vaccine composition	Formulation	Storage conditions	Number of doses	Schedule
Rotarix, RV1; GSK	Globally	March 2009	Live-attenuated, human wild-type G1P[8] strain [R1X4414]	Liquid	2–8°C for 36 months	2	2 and 4 months
RotaTeq RV5; Merck	Globally	October 2008	Live-attenuated, human-bovine rotavirus reassortant G1, G2, G3, G4, and P[8]	Liquid	2–8°C for 36 months	3	2, 4, and 6 months
Rotavac Bharat	Globally	January 2018	Live-attenuated wild-type reassortant G9P[11] strain [116E]	Liquid frozen	2–8°C for 7 months, 20°C (long-term)	3	6, 10, and 14 weeks
Rotasiil serum institute	Globally	September 2018	Live-attenuated human-bovine rotavirus reassortant G1, G2, G3, G4, and G9	Lyophilized, thermostable lyophilized, and liquid	<40°C for 18 months <25°C for 30 months 14 weeks	3	6, 10, and 14 weeks

Table is composed by following references (Burnett et al., 2018; Burke et al., 2019; Glass et al., 2021; Omatola and Olaniran, 2022b).

## Rotavirus vaccine implementation

In 2008, prior to the widespread adoption of rotavirus vaccines, RVGE (Rotavirus Gastroenteritis) resulted in approximately 453,000 fatalities worldwide among children under the age of 5 (Tate et al., 2012). In 2009, the World Health Organization (WHO, 2013) advised that all nations should incorporate rotavirus vaccines into their national immunization programs (NIPs). The WHO emphasized the need to prioritize the introduction of rotavirus vaccines in regions of South and South East Asia and sub-Saharan Africa, where mortality rates associated with rotavirus gastroenteritis (RVGE) are notably high (WHO, 2013). As of November 2019, rotavirus vaccination had been implemented or was in the planning stages in 120 countries, with 98 of them integrating the vaccine into their immunization programs, whether through universal immunization programs, phased approaches, public funding, or organizations (GAVI, 2023; WHO, 2023). It is worth noting that the majority of countries that have introduced rotavirus vaccination fall within the middle- and high-income categories, or they are eligible for support from the Global Alliance for Vaccines and Immunization (GAVI) (Buchy et al., 2021). Despite the WHO’s strong recommendations, the adoption of rotavirus vaccines into NIPs has been considerably lower in Asian countries compared to those in Africa. In the regions of Asia and the Pacific, the Southeast Asia region (SEAR) reports the lowest vaccination coverage at 18.0%, followed by the Western Pacific region (WPR) at 30.0%. This disparity suggests that these regions are lagging in their efforts to combat RVGE, despite the substantial burden it places on their populations (WHO, 2023). With the introduction of rotavirus vaccination, it is estimated that RVGE-related deaths decreased to 146,500 in 2015 and further dropped to 128,500 in 2016 (Troeger et al., 2018b). Despite the decrease in RVGE-related deaths in the post-vaccination era, there still exists a significant overall disease burden, both directly and indirectly, due to the high number of RVGE cases, hospitalizations, and associated complications. In 2016, rotavirus infections were responsible for an estimated 258 million instances of diarrhea, and RVGE led to 1,537,000 hospitalizations globally among children under 5 years old. The majority of this burden is concentrated in low-income regions, particularly in Africa and Asia (Troeger et al., 2018b).

## Post vaccine effects in developing countries

Rotavirus vaccines have been shown to be highly effective in reducing the burden of the disease, even in LMICs. Studies conducted in East Africa have reported a notable decrease in hospital admissions, ranging from 40 to 70% (Bar-Zeev et al., 2015; Abeid et al., 2017; Mujuru et al., 2019), and a reduction of severe diarrhea attributed to rotavirus, ranging from 39 to 61%. Furthermore, a recent amalgamation of data from the sub-Saharan Africa region has revealed a noteworthy decline, albeit with some internal variations, in the prevalence of rotavirus-positive cases. This reduction has been observed to decrease from 42% during the pre-vaccination period to 21% in the post-vaccination period (Kabayiza et al., 2023). As rotavirus vaccines become integrated into immunization programs across low-income countries worldwide, it becomes crucial to assess the real-world impact of vaccination. This assessment is essential for gaining a deeper understanding of vaccine effectiveness and safety in various contexts. While both licensed rotavirus vaccines have demonstrated strong protection against a variety of circulating rotavirus strains, including those with G and P types not covered by the vaccine, continuous monitoring of the long-term effects of vaccination on strain ecology remains critical. Additionally, given the moderate efficacy of rotavirus vaccines in low-income countries, it is important to consider and evaluate interventions aimed at enhancing vaccine performance, such as administering additional vaccine doses or exploring alternative vaccination schedules. Furthermore, to maintain the global implementation of vaccination, it is imperative to ensure a consistent and affordable supply of rotavirus vaccines. It is heartening to note that several manufacturers in emerging markets like India, China, Indonesia, and Brazil are developing potential rotavirus vaccines that may become accessible within the next 5 years (Crawford et al., 2017).

While the introduction of vaccines has lessened the impact of rotavirus, heightened immune pressure from vaccination against natural rotavirus strains could lead to the emergence of immune escape mutants or strains with different antigens. As a result, it is crucial to conduct post-vaccine surveillance of circulating rotavirus genotypes. This surveillance helps track shifts in transmission patterns, identify newly generated genotypes due to vaccination (such as neutralization escape mutants), and uncover potentially harmful strains associated with the vaccine (Zhuo et al., 2023).

## Causes of delayed vaccine introduction

The causes behind the delayed introduction of vaccines vary from one country to another. Different aspects of the implementation process pose challenges, such as collecting evidence, decision-making procedures, securing funding, planning, and executing the rollout. In certain countries, the primary reason for not adopting rotavirus vaccination might be the belief that it offers limited advantages, while in others, economic considerations like the cost-effectiveness of rotavirus vaccination programs take precedence. Other commonly mentioned factors contributing to delayed vaccine introduction include the absence of country-specific data on the efficacy and safety of the vaccine, as well as its impact on reducing rotavirus-related illnesses. These factors are essential for verifying the clinical protection offered by rotavirus vaccination against RVGE. Furthermore, having high-quality epidemiological data is critical for informing governments that are contemplating the inclusion of rotavirus vaccination in their National Immunization Programs (NIPs) (Kirkwood and Steele, 2017).

## Increasing cold chain capacity

Despite favorable cost-effectiveness analyses and pricing, numerous countries planning to introduce a rotavirus vaccine might encounter obstacles stemming from insufficient cold-chain storage capabilities. The vaccine’s substantial packaging volume presents a logistical challenge, as many countries lack the necessary space for its storage upon implementation (Babji and Kang, 2012). Unfortunately, the regions most burdened by rotavirus disease tend to be those with the least capacity for storing and administering the vaccine effectively. Therefore, it is imperative to assess cold-chain storage capacity and resource requirements before procuring the vaccine to prevent wastage. Ongoing efforts should prioritize expanding cold-chain capacity in developing nations and conducting comprehensive assessments of a country’s capabilities before introducing the vaccine. It is worth noting that this issue could potentially be alleviated through the further development and production of heat-stable vaccines, such as the one created by the Serum Institute of India, which remains stable for up to 2 years at 37°C or 6 months at 40°C (De Oliveira et al., 2008; Hallowell et al., 2020).

## Enhancing vaccine effectiveness in less developed regions

The effectiveness of the rotavirus vaccine and the duration of immunity in developing countries are approximately half of what is observed in developed countries. The good news is that there are various potential interventions to consider, such as staggering the administration of poliovirus and rotavirus vaccines, offering catch-up doses, transitioning from oral vaccination to microneedle skin patch vaccinations, and exploring parenteral vaccination methods. Since more than 85% of the world’s children reside in developing countries, even implementing interventions that yield minor enhancements in vaccine performance could have a profound impact on reducing the global burden of rotavirus disease. These improvements in vaccine performance also have the potential to maintain the cost-effectiveness of the rotavirus vaccine as the primary solution for preventing rotavirus disease (Moon et al., 2013; Burke et al., 2019).

## The future control of RVA diarrhea

In the nearly five decades since the discovery of rotavirus, there has been a significant reduction in childhood mortality due to diarrhea. In 1986, there were approximately 3.6 million deaths, which decreased to around 500,000 in 2018. Much of this improvement occurred in the approximately 35 years before the introduction of rotavirus vaccines. Mathematical models suggest that these declines in mortality in low-income countries can be attributed to various factors, including improved treatment with rehydration therapy, increased breastfeeding, better birth-spacing, maternal education, delayed pregnancies, smaller family sizes, and advancements in water, sanitation, and hygiene.

Since 2009, the World Health Organization (WHO) has recommended the use of rotavirus vaccines in all countries, and GAVI has subsidized vaccine purchases for low-income countries. Since 2018, two new Indian manufacturers have significantly increased production, making rotavirus vaccines available to approximately 54% of the world’s children in about 100 countries. This has led to a major decrease in the global burden of severe rotavirus disease. By 2018, estimates of annual rotavirus-related deaths had decreased to approximately 150,000–200,000, and the percentage of severe diarrhea attributed to hospitalizations had dropped from approximately 34–40% to 20–24%, with variations by country.

To further reduce the burden of rotavirus diarrhea in the future, several strategies are needed. Firstly, rotavirus vaccines must be introduced in approximately 90 countries, which collectively host around 46% of the world’s children, and where rotavirus immunization has not yet been implemented into national immunization programs. This will require policymakers to assess the local disease burden and determine the cost threshold at which implementing a national program becomes feasible. Manufacturers also need to work on reducing vaccine costs as production volume increases. Simultaneously, ongoing research is focusing on developing the next generation of parenteral or skin patch rotavirus vaccines that could be more effective and potentially replace the oral vaccines. If these vaccines prove to be safe, more efficient, easier to administer as part of a combined vaccine, and affordable for all, they could contribute to achieving full control over the disease (Glass et al., 2021).

The vision of controlling rotavirus diarrhea to improve the health and survival of all children has evolved significantly since the virus’s discovery in 1973. From a global aspiration, we have now reached a point where four globally licensed vaccines are routinely used in over 100 countries. However, low-income countries that have introduced rotavirus vaccination into their national programs still face rotavirus as a significant cause of diarrheal hospitalizations and deaths. Therefore, continued efforts to develop more effective vaccines are essential to ultimately control this disease worldwide, especially in countries with high mortality rates from diarrhea, where these vaccines are needed the most.

In LMICs, where conditions often favor the rapid spread of infectious diseases, targeted efforts to enhance sanitation can have a profound impact. Implementing proper waste disposal systems, ensuring access to clean and safe drinking water, and promoting the construction and use of sanitary facilities are crucial steps in preventing the transmission of rotavirus. Good hygiene practices, such as regular hand washing with soap and water, also contribute significantly to the reduction of rotavirus infections. Educating communities about the importance of hand hygiene, especially before meals and after using the toilet, can empower individuals to protect themselves and others from the virus. Additionally, public health campaigns that focus on raising awareness about the transmission pathways of rotavirus and the benefits of improved sanitation and hygiene can foster positive behavioral changes. By addressing the root causes of infection, these measures not only reduce the incidence of rotavirus but also contribute to overall community well-being.

## Continuing studies on rotavirus vaccines

In particular, for low- and middle-income countries (LMICs) with a high rate of disease transmission resulting in an early disease burden, initiating immunization schedules right from birth is anticipated to offer additional advantages compared to existing schedules. These advantages include providing earlier protection, improving vaccine coverage rates, and potentially enhancing safety, as intussusception is a rare occurrence among newborns (Bines et al., 2018). A novel oral rotavirus vaccine known as RV3-BB, developed from a distinct neonatal strain G3P [6] in Australia, has displayed promising results. A Phase 2b clinical trial conducted with neonates in Indonesia demonstrated similar vaccine efficacy when administered according to a neonatal schedule, given at birth, followed by doses at 8 and 14 weeks, in comparison to the standard infant schedule (8, 14, and 18 weeks) (Bines et al., 2018). These encouraging findings were recently reinforced by a study conducted in New Zealand using the same vaccine in neonates. If the neonatal schedule proves advantageous, a similar approach might be applied to Rotavac, which is based on a neonatal strain identified in India, especially in countries with high child mortality rates. Another strategy to enhance vaccine performance involves the development of injectable rotavirus vaccines, inspired by the inactivated polio vaccine model, to address the lower effectiveness of oral vaccines in LMICs. The leading candidate in this category is the trivalent subunit vaccine P2-VP8*, originally discovered at the National Institutes of Health (NIH) and currently undergoing Phase 3 efficacy trials in multiple African countries, following successful Phase 1 and 2 studies, under the supervision of PATH (Groome et al., 2017, 2020). Non-replicating formulations of already licensed oral vaccines, mRNA vaccines, virus-like particle vaccines, and nanoparticle vaccines are also in early stages of development (Lee, 2021). Injectable vaccines are expected to exhibit superior effectiveness as they bypass the previously mentioned factors that have led to suboptimal oral vaccine efficacy (Patel et al., 2009; Steele et al., 2019). Additionally, they offer advantages such as an improved safety profile, reduced cold-chain requirements, lower costs, and the potential for co-formulation with other parenterally administered vaccines. Nevertheless, their deployment may still depend on various factors.

## Future directions and policy recommendations

Future directions in the management of rotavirus disease and the promotion of rotavirus vaccines should focus on a multifaceted approach to reduce the global burden of this highly contagious and potentially life-threatening infection. First and foremost, enhancing vaccine coverage remains a priority. Governments and international organizations should collaborate to ensure that rotavirus vaccines are accessible and affordable for all children, particularly in low- and middle-income countries where the disease has the greatest impact. Continued research and development efforts should aim to improve vaccine formulations, increase their efficacy, and extend the duration of protection. Additionally, ongoing surveillance and epidemiological studies are essential to monitor the changing landscape of rotavirus strains and to inform vaccine updates when necessary. In terms of policy recommendations, governments should prioritize inclusion of rotavirus vaccines in their national immunization programs and provide robust education campaigns to raise awareness about the importance of vaccination. Furthermore, healthcare systems should be strengthened to ensure timely diagnosis and treatment of rotavirus infections, reducing the associated morbidity and mortality. Ultimately, a global commitment to comprehensive vaccination strategies and public health measures will be crucial in the fight against rotavirus disease.

## Conclusion

In conclusion, the review article delves into the multifaceted landscape of rotavirus in developing countries, providing a comprehensive exploration of its molecular diversity, epidemiological nuances, and the crucial strategies needed for effective vaccination. By scrutinizing the intricate variations in rotavirus strains, the article highlights the challenges posed by this infectious agent and the necessity for region-specific approaches to vaccination. The epidemiological insights presented shed light on the dynamic nature of rotavirus transmission in developing countries, emphasizing the need for targeted interventions to curb its impact on vulnerable populations. Furthermore, the review underscores the significance of vaccination as a pivotal tool in mitigating the burden of rotavirus-related morbidity and mortality. As we navigate the complexities of implementing vaccination programs in resource-constrained settings, the article advocates for a holistic approach that considers not only the scientific aspects but also the socio-economic and infrastructural challenges unique to each region. It emphasizes the importance of collaboration between healthcare providers, policymakers, and the global community to ensure the successful implementation and sustainability of vaccination initiatives.

## Author contributions

AS: Conceptualization, Data curation, Formal analysis, Funding acquisition, Investigation, Project administration, Resources, Software, Validation, Visualization, Writing – original draft, Writing – review & editing. JK: Writing – review & editing.
